# Splice modulating antisense oligonucleotides restore some acid-alpha-glucosidase activity in cells derived from patients with late-onset Pompe disease

**DOI:** 10.1038/s41598-020-63461-2

**Published:** 2020-04-21

**Authors:** May Thandar Aung-Htut, Kristin A. Ham, Michel Tchan, Russell Johnsen, Frederick J. Schnell, Sue Fletcher, Steve D. Wilton

**Affiliations:** 1 Centre for Molecular Medicine and Innovative Therapeutics, Murdoch University, Murdoch, 6150 Australia; 2 Genetic Medicine, Westmead Hospital, Sydney, 2145 Australia; 3 Sydney Medical School, The University of Sydney, Sydney, 2006 Australia; 4 Sarepta Therapeutics, Cambridge, 02142 USA; 5 Perron Institute for Neurological and Translational Science and Centre for Neuromuscular and Neurological Disorders, The University of Western Australia, Perth, 6009 Australia

**Keywords:** Metabolic disorders, Genetics, Molecular biology, Molecular medicine

## Abstract

Pompe disease is caused by mutations in the *GAA* gene, resulting in deficient lysosomal acid-α-glucosidase activity in patients, and a progressive decline in mobility and respiratory function. Enzyme replacement therapy is one therapeutic option, but since not all patients respond to this treatment, alternative interventions should be considered. One *GAA* mutation, c.-32-13T > G, impacts upon normal exon 2 splicing and is found in two-thirds of late-onset cases. We and others have explored a therapeutic strategy using splice modulating phosphorodiamidate morpholino oligomers to enhance *GAA* exon 2 inclusion in the mature mRNA of patients with one c.-32-13T > G allele. We designed 20 oligomers and treated fibroblasts derived from five patients to identify an oligomer sequence that maximally increased enzyme activity in all fibroblasts. The most effective splice correcting oligomer was chosen to treat forced-myogenic cells, derived from fibroblasts from nine patients carrying the c.-32-13T > G mutation. After transfection, we show increased levels of the full-length *GAA* transcript, acid-α-glucosidase protein, and enzyme activity in all patients’ myogenic cells, regardless of the nature of the mutation in the other allele. This data encourages the initiation of clinical trials to assess the therapeutic efficacy of this oligomer for those patients carrying the c.-32-13T > G mutation.

## Introduction

Intronic variations that lead to aberrant splicing events, such as exon loss or the retention of intronic sequence in the mature mRNA in the form of pseudo-exons, have been reported in many genes^1,2^. Most recently, Milasen, a splice switching compound targeted to the *CLN7* pseudo-exon mutation in one Batten disease patient, was granted approval by the US Food and Drug Administration^3^. There is growing interest in the use of splice switching antisense oligonucleotides (AOs) as therapeutic agents to treat serious inherited diseases. At present, three splice switching AOs, Vyondys 53^4^, Exondys 51^5^, and Spinraza^6^, have been approved by the US Food and Drug Administration as treatments for a subset of patients with Duchenne muscular dystrophy and spinal muscular atrophy, respectively.

The late-onset form of Pompe disease, also known as glycogen storage disease type II (GSD II), presents as a suitable candidate for AO therapy, since approximately two-thirds of the adult Pompe patients harbour a common disease-causing mutation: c.-32-13T > G^7^. The incidence of this variant is higher in Caucasians and identified in ninety percent of the adult-onset Pompe patients^8^. This mutation is known to cause complete skipping of exon 2 from most *GAA* transcripts (Supplementary Fig. S1)^9,10^, and disease onset and severity is modestly correlated with the residual lysosomal acid-α-glucosidase (GAA) activity in those patients^11–13^. Generally, less than 1% of normal GAA activity is observed in those presenting with the infantile form of the disease. Juvenile-onset patients generally have less than 10% GAA activity, while less than 30% activity is observed in adult-onset patients.

Since Pompe disease arises from an insufficiency of the GAA enzyme, enzyme replacement therapy (ERT) is one therapeutic option. Intravenous administration of recombinant human GAA, Lumizyme (alglucosidase alfa, also marketed as Myozyme), manufactured by Sanofi-Genzyme, Framingham, MA^14^, shows modest responses with limited efficacy in mitigating muscle weakness and respiratory dysfunction^15,16^ and 25% of patients may not respond to the treatment^17^. Consequently, the second generation of recombinant GAA, avalglucosidase alfa, with increased mannose 6-phosphate residues to enhance GAA uptake was developed^18^. A phase 1 study on safety, pharmacokinetic and pharmacodynamic of avalglucosidase alfa in late-onset Pompe patients showed that the enzyme was well-tolerated, however anti-avalglucosidase alfa antibodies were detected in 90% of the patients who have not previously received ERT^19^. In addition, the first human, open-label, phase 1/2 trial for combination therapy of a modified GAA, in conjunction with a small molecule pharmacological chaperone, has also been initiated (NCT02675465, https://clinicaltrials.gov).

Gene replacement therapy by intra-diaphragmatic injections of an adeno-associated viral vector encoding the human *GAA* cDNA has been evaluated^20^. However, immune responses against the viral capsid protein and transgene were detected in these patients. While the development of antibodies against the viral capsid is a major drawback of gene therapy, co-administration of an an immunosuppressive regimen and the vector carrying the *GAA* transgene is currently being investigated (NCT02240407, https://clinicaltrials.gov), as are many other strategies to improve gene therapy for Pompe disease (for detail review see^21^). Consequently, there is a strong justification for the investigation and evaluation of alternative therapies.

We have extensive experience in designing splice switching AOs, including those to treat Duchenne muscular dystrophy (DMD)^22^ and spinal muscular atrophy^23,24^. We designed and tested numerous AOs to prevent aberrant *GAA *exon 2 splicing and enhance normal processing of the *GAA* transcript in late-onset Pompe patient-derived fibroblast cell strains carrying the common c.-32-13T > G mutation (subsequently published in the patent application WO2015035231 A1, 5 September 2014). van der Wal *et al*. also reported AO sequences that prevent aberrant exon 2 splicing in patients with the c.-32-13T > G mutation using a vectorised *U7* snRNA AO expression system^25,26^. Using a minigene approach and fibroblasts derived from a patient possessing the c.-32-13T > G mutation, Goina *et al*. were able to correct exon 2 splicing and rescue GAA enzyme production using a combination of three morpholinos targeting splicing silencers within *GAA* exon 2^27^. Here, we report AO sequences that showed consistent improvement in GAA activity in MyoD-induced myogenic cells derived from nine patients.

The well-established PMO safety profile shown in DMD patients who have received weekly infusions of a PMO over nine years^28,29^ supports further development of a PMO splice switching intervention for late-onset Pompe disease. Since these patients have already been exposed to full-length GAA and no exogenous proteins are being administered, immunogenic responses are not anticipated.

## Results

### **Characterisation of patients-derived fibroblasts**

The Pompe patient fibroblasts GM00443 and GM11661, purchased from Coriell Cell Repositories are designated as C 1 and C 2, respectively and those from Westmead Hospital, Sydney, Australia as WM 1 to 9. The patient cell strains, WM 3 and 6, were not included in this study as these cells either did not survive or had a considerably reduced proliferation rate (Table 1).

**Table 1:** Patient cell strain summary.

Patient ID	Source	Our ID	Mutations		Remark
			Allele 1	Allele 2	
GM00443	Coriell	C 1	c.-32-13T > G	c.2608C > T (p.Arg870X)	c.2608C > T causes NMD
GM11661	Coriell	C 2	c.-32-13T > G	Genomic deletion of exon 18	
Patient 1	Westmead	WM 1	c.-32-13T > G	c.2074C > T (p.Gln692X)	c.2074C > T causes NMD
Patient 2	Westmead	WM 2	c.-32-13T > G	c.1910_1918del (p.Leu637_Val639del)	
Patient 4	Westmead	WM 4	c.-32-13T > G	c.1128_1129delinsC (p.Tyr376CysfsX16)	c.1128_1129delinsC cause intron 6 inclusion, NMD
Patient 5	Westmead	WM 5	c.-32-13T > G	c.1548G > A (p.Trp516X)	c.1548G > A causes NMD
Patient 7	Westmead	WM 7	c.-32-13T > G	c.953T > C (p.Met318Thr)	
Patient 8	Westmead	WM 8	c.-32-13T > G	c.1128_1129delinsC (p.Tyr376CysfsX16)	c.1128_1129delinsC cause intron 6 inclusion, NMD
Patient 9	Westmead	WM 9	c.-32-13T > G	c.1082C > G (p.Pro361Arg)	

**Figure 1:**
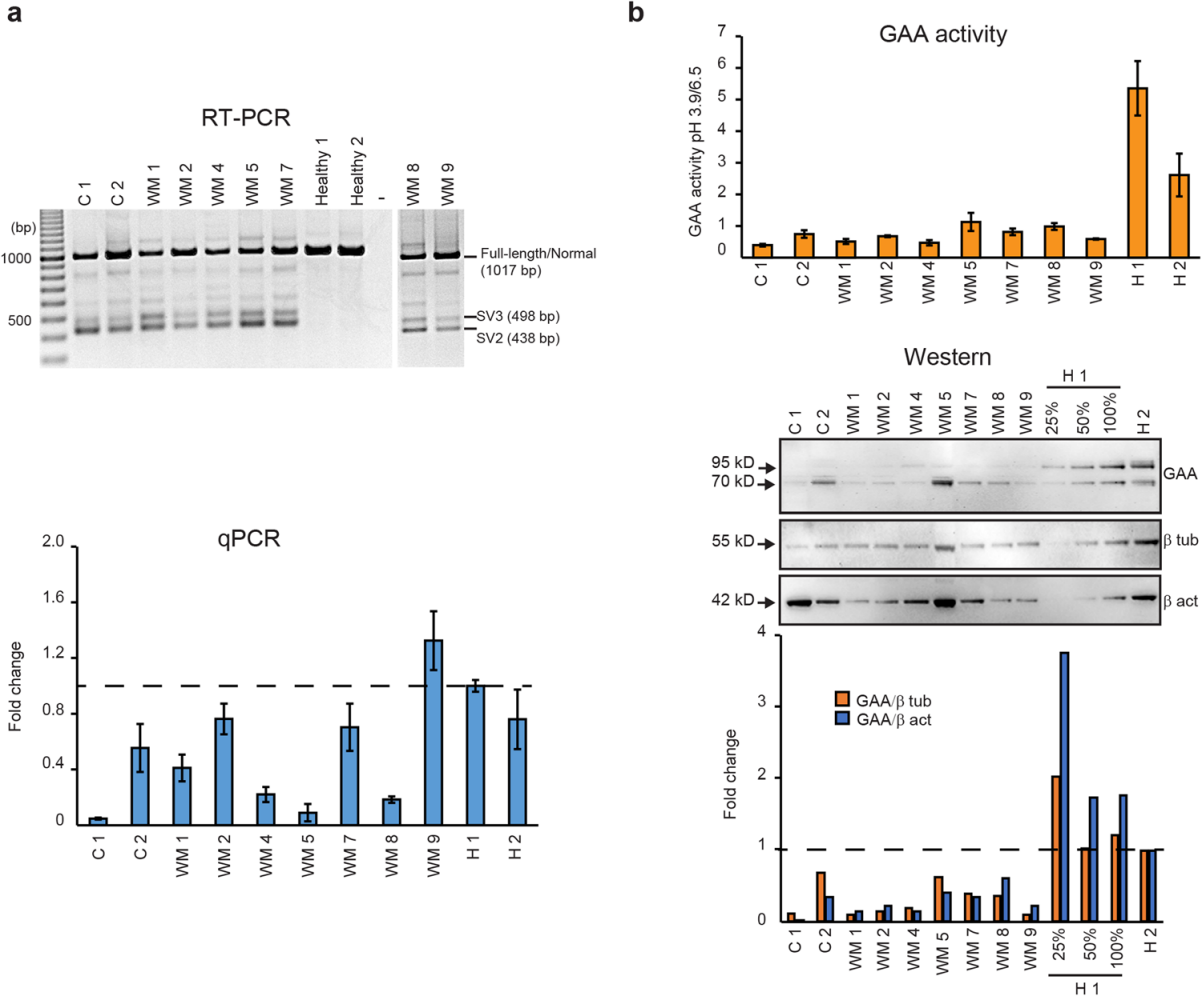
*GAA* transcript and GAA protein expression and activity of nine Pompe patients compared to two healthy control fibroblasts. (**a**) RT-PCR products of *GAA* transcript amplified from exon 1 to 5, and qPCR analysis of *GAA* transcript with full-length exon 2 using RNA isolated from fibroblasts derived from patients (C 1, C 2, WM 1, WM 2, WM 4, WM 5, WM 7, WM 8 and WM 9) and two healthy individuals (H 1 and H 2). The qPCR analysis was performed as the relative expression of *GAA* transcript compared to *TBP* transcript, encoding TATA-box binding protein, and is presented as the mean fold change ±SDs of triplicates compared to H 1 (healthy 1). SV2/3; splice variant 2/3. −; no template control. (**b**) GAA activity (mean ± SDs of at least three biological replicates) and protein found in nine patients and two healthy individuals derived fibroblasts. β tub; beta-tubulin, β act; beta-actin. The densitometric analysis of the western blot is shown as a bar graph below the Western image. The gels were cropped for presentation, and full-size gels are presented in Supplementary Fig. S5.

We confirmed the c.-32-13T > G mutation on one allele in all patients, and the previously known second mutations for patient C 2, WM 1, 4, and 7 (Table 1 and Supplementary Fig. S1). We identified the second mutations for patients, C 1, WM 2, 5, 8, and 9 (Table 1 and Supplementary Fig. S1), as they were not previously characterised. Nonsense mutations, likely to induce nonsense-mediated decay (NMD) of the *GAA* transcripts, were found in patients C 1, WM 1, WM 4, WM 5 and WM 8. Lower levels of the full-length *GAA* transcript were consistently evident in these patients after amplification across exons 1 to 5 using RT-PCR and qPCR analysis (Fig. 1a). Highly efficient NMD of the *GAA* transcripts was apparent since these nonsense mutations were not found by DNA sequencing of the RT-PCR amplicons derived from the patients. Cryptically spliced SV2 (missing entire exon 2) and SV3 (partial exon 2) transcripts were present in all patients-derived fibroblasts. Due to the GC-rich nature of the template, additional unidentified bands, reported by others^27^ were also present. We found that the c.1128_1129delinsC mutation promotes inclusion of part of intron 6 in 20-30% of the mature *GAA* transcripts (Supplementary Fig. S1) from cell lines WM 4 and 8, leading to a stop codon after p.Gly359. We also confirmed an in-frame deletion of exon 18 from the *GAA* transcript in patient C 2 (Supplementary Fig. S1).

Correctly spliced normal *GAA* transcript with full exon 2 inclusion was quantitated using qPCR for all untreated fibroblasts from patients and two healthy individuals, H 1 and H 2 (Fig. 1a). The total functional full-length *GAA* transcript expression varies between the two healthy fibroblasts. All the patient-derived fibroblasts (C 1, WM 1, 4, 5 and 8) carrying nonsense mutations showed lower levels of the full-length *GAA* transcript than those carrying the in-frame deletions or missense mutation (C 2, WM 2, 7 and 9).

The residual GAA activity in patient-derived fibroblasts was analysed and compared to that measured in fibroblasts from H1 and H2 (Fig. 1b). GAA activity in the H 2 cells was approximately half that in H 1 cells, which is consistent with the lower *GAA* transcript expression determined by qPCR. In this study, the GAA activity in patient-derived fibroblasts was less than 40% of that assayed in H 2 cells. Analysis of GAA protein by western blotting supports the observation that all patient-derived fibroblasts have lower GAA protein levels than the two healthy individuals (Fig. 1b).

### Antisense oligonucleotide design and treatment in fibroblasts

**Figure 2:**
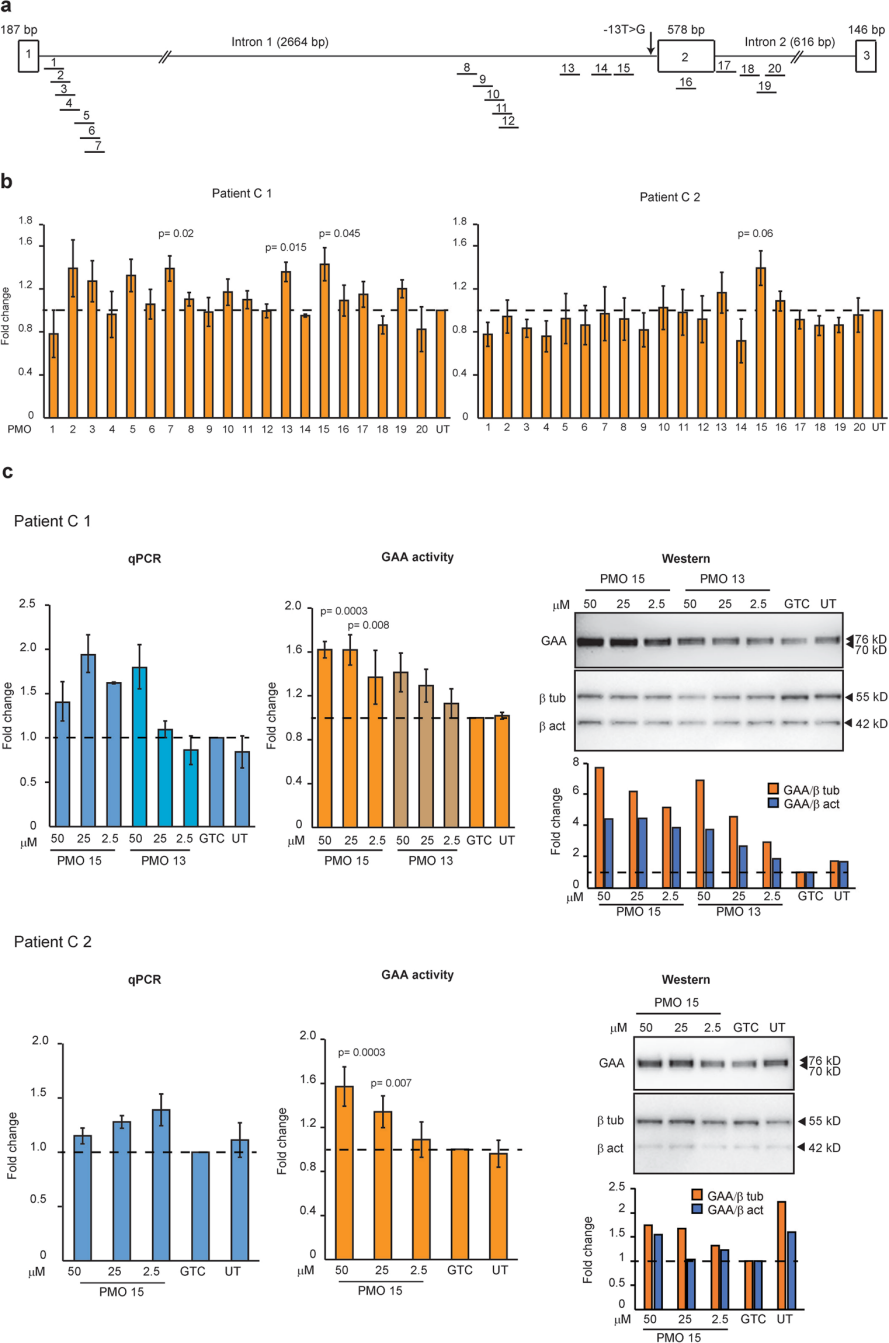
Preliminary screening for the best PMO sequences that increased GAA activity in patientsderived fibroblasts. (**a**) The relative annealing positions of the AOs synthesised as PMOs are shown. PMOs are indicated as solid lines with the corresponding number shown above. Exons are shown as boxes and introns as lines (not drawn to scale). (**b**) The improvement of GAA activity in treated compared to untreated patient C 1 and C 2 derived fibroblasts. Data represent the mean fold change ±SDs of two biological replicates (n = 2). (**c**) qPCR, GAA enzyme activity (average fold change ±SDs of two biological replicates) and western analysis of patient C 1 and C 2 fibroblasts treated with different dosages of PMO 13, 15 and GTC (Gene Tools Control). UT; untreated. β tub; beta-tubulin, β act; beta-actin. The densitometric analysis of the western blot is shown as a bar graph below the Western image. The gels were cropped for presentation, and full-size gels are presented in Supplementary Fig. S5. GAA assays were performed in triplicate.

In order to identify splice motifs involved in exon 2 selection during pre-mRNA processing of the c.-32-13T > G *GAA* transcript, we analysed *GAA* intron 1, exon 2 and intron 2 using SpliceAid 2^30^. Several AOs targeting the predicted binding motifs for hnRNP H and I, and MBNL1, known to mediate exon exclusion, were designed (Table 2, Fig. 2a and Supplementary Fig. S2). The PMO sequences 1-3, 4-7, 8-12 and 18-20 partially overlap. The PMOs were delivered into patient fibroblasts (C 1, C2, WM 1, WM 2, and WM 4) using nucleofection. GAA enzyme activity assays were performed in triplicate, four days after PMO treatment, to identify oligomers that promote *GAA* exon 2 retention in the mature transcript (Fig. 2b and Supplementary Fig. S2).

**Table 2:** Coordinates and sequences of AOs used in this study.

Name	Coordinates	Sequences (5′-3′)
PMO 1	c.-33 (+6 + 27)	GCG GGG CAG AGC TCA GGT GT
PMO 2	c.-33 (+10 + 29)	CAG CGC GGG GCA GAC GTC AG
PMO 3	c.-33 (+14 + 33)	CCG GCA GCG CGG GGC AGA CG
PMO 4	c.-33 (+17 + 36)	CCG CCG GCA GCG CGG GGC AG
PMO 5	c.-33 (+24 + 43)	GAT GTT ACC GCC GGC AGC GC
PMO 6	c.-33 (+28 + 47)	CTG GGA TGT TAC CGC CGG CA
PMO 7	c.-33 (+32 + 51)	GCT TCT GGG ATG TTA CCG CC
PMO 8	c.-32 (−509-490)	TCT CGA ACT CCT GAG CTC AA
PMO 9	c.-32 (−500-481)	CCA GGC TGG TCT CGA ACT CC
PMO 10	c.-32 (−487-468)	TTT GCC ATG TTA CCC AGG CT
PMO 11	c.-32 (−480-461)	ACG GGA TTT TGC CAT GTT AC
PMO 12	c.-32 (−475-456)	TAG AGA CGG GAT TTT GCC AT
PMO 13	c.-32 (−179-160)	GAG AGG GCC AGA AGG AAG GG
PMO 14	c.-32 (−99-75)	GAC ATC AAC CGC GGC TGG CAC TGC A
PMO 15	c.-32 (−74-55)	GGC TCT CAA AGC AGC TCT GA
PMO 16	c.170_194	GGC CCT GGT CTG CTG GCT CCC TGC T
PMO 17	c.546 (+14 + 30)	TGG CCG CCG CCC CCG CCC
PMO 18	c.546 (+40 + 59)	TGT CGA TGT CCA CGC GCA CC
PMO 19	c.546 (+53 + 72)	GTG AGG TGC GTG GGT GTC GA
PMO 20	c.546 (+67 + 86)	GCA CCC CAC CCT TGT GAG GT

Treatment with PMO 15 consistently increased GAA activity in three different patient-derived fibroblast strains, while PMO 5 and 13 also increased GAA activity in two or more patient cell strains. Since many PMOs failed to increase GAA activity, any improvement in GAA activity must be attributed to the PMO treatment. PMOs 13 and 15 were further titrated to demonstrate concentration-dependent effects in patient C 1 and PMO 15 in C 2 fibroblasts (Fig. 2c). A control PMO sequence (GTC) from Gene Tools was included in all experiments as a sham transfection experimental control. An apparent dose-dependent increase in GAA activity was observed in both patient-derived fibroblasts, with a better response to PMO 15 seen in C 1 fibroblasts compared to C 2. Additional analyses were performed to confirm that the elevated GAA activity correlated with changes in full-length *GAA* transcript and GAA protein expression, and it was the case for both patient cell strains. Both beta-tubulin and beta-actin were analysed and used as reference loading controls on western blots. Nucleofection with the GTC PMO caused a slight knockdown in GAA protein expression in both cell strains, possibly due to the nucleofection process, although overall GAA activity was not significantly altered compared to the untreated samples. Neither PMO 13 nor 15 had a significant effect on GAA activity and *GAA* transcript when nucleofected into fibroblasts derived from H 1, however a slight decrease in GAA protein level was observed after PMO 15 treatment (Supplementary Fig. S2).

### Antisense oligonucleotide treatment in myogenic cells

**Figure 3:**
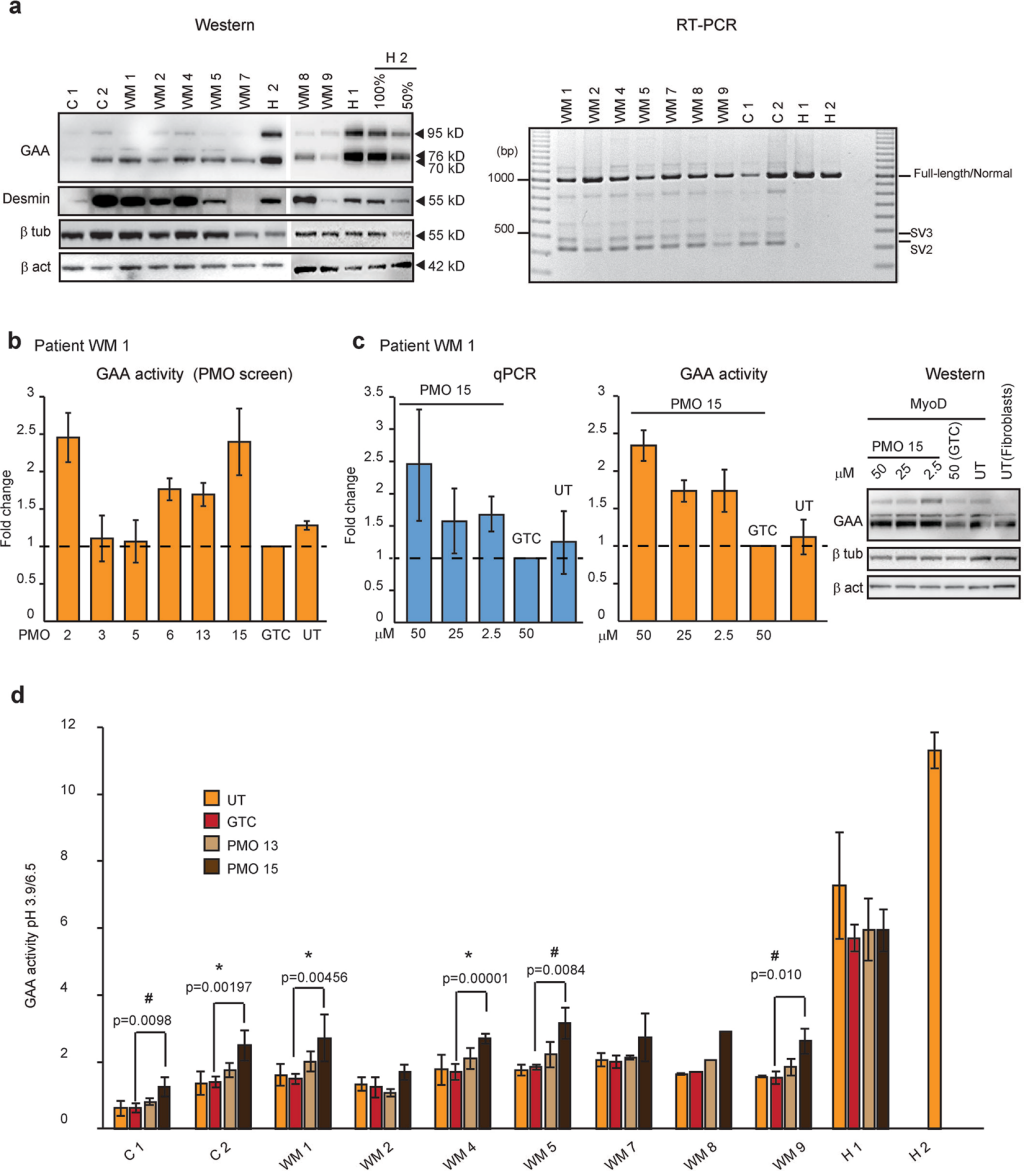
The efficiencies of selective PMOs in myogenic cells derived from the patient after forced myogenesis. (**a**) Western analysis of muscle-specific protein desmin and RT-PCR analysis of *GAA* transcript (exon 1 to 5) in patients’ and healthy controls’ fibroblasts forced into the myogenic lineage by infection with MyoD expressing virus. β tub; beta-tubulin, β act; beta-actin. (**b**) GAA activity of patient WM 1 forced myogenic cells after treatment with various PMOs. Data represent the mean ± SDs of triplicates. (**c**) qPCR analysis of *GAA* transcript with exon 2 (the mean ± SDs of triplicates), GAA activity (the mean ± SDs of triplicates) and GAA protein in patient WM 1 forced myogenic cells after treatment with various dosages of PMO 15, Gene Tools Control (GTC) and untreated (UT). (**d**) Comparison of GAA activity in nine patients’ and two healthy controls’ (H 1 and H 2) forced myogenic cell cultures after treatment with PMO 13, 15 and GTC at 50 µM and untreated (UT) samples. Data represent the mean ± SDs of at least three biological replicates (n ≥ 3) for patient C 1, C 2, WM 1, 2, 4, 5, 7, and 9. The difference of the means, compared to GTC is significant at *p < 0.05 and ^#^p < 0.1 using ANOVA with Bonferroni correction. No biological replicates were performed for WM 8. The gels were cropped for presentation, and full-size gels are presented in Supplementary Fig. S5.

A characteristic feature of Pompe disease is the accumulation of glycogen in skeletal muscle. Therefore, the PMOs that induced enhanced GAA activity in patient-derived fibroblasts were also evaluated in fibroblasts forced into the myogenic lineage by transduction with a MyoD expressing adenovirus (Fig. 3)^31^.

Variable levels of the muscle-specific protein desmin were detected in most patient-derived myogenic cells, indicating transformation of fibroblasts into the myogenic lineage (Fig. 3a), except for the WM 7 cell strain. The levels of desmin protein were low in MyoD induced C 1 and WM 9 cells compared to the other MyoD induced cell strains. Total GAA protein found in all patient-derived myogenic cells was generally less than 50% of that present in H 2. We also confirmed the presence of *GAA* transcript isoforms SV2 and 3 in all patient myogenic cells (Fig. 3a), affirming myogenic conversion did not alter the *GAA* spliceoforms identified in the fibroblasts.

We then treated myogenic cells derived from patients C1, C 2, WM 1, WM 2 and WM 4 with PMO 2, 3, 5, 6, 13 and 15, selected according to the outcome of the AO screen in fibroblasts. Activity of GAA was analysed six days after PMO delivery into these myogenic cells (Fig. 3b and Supplementary Fig. S3). PMO 15 increased GAA activity in all the patient cells tested. The greatest improvement was observed in patient cell strains WM 1 and C 1, with approximately 2.5 fold increase in GAA activity. (Supplementary Fig. S3).

To further confirm PMO 15 as the lead candidate in correcting *GAA* transcript expression and increasing functional protein levels, patient-derived myogenic cells were treated with different concentrations of PMO 15 and *GAA* transcript, GAA activity and protein levels were analysed (Fig. 3c and Supplementary Fig. S4). A dose-dependent increase in GAA activity was observed in all patients’ myogenic cells, and an increase in full-length *GAA* transcript retaining exon 2 was also verified. For western analysis, both beta-tubulin and beta-actin were used as loading controls. However, in some patients’ myogenic cells (patient WM 2, WM 7 and WM 9) the relative expression levels of beta-tubulin to beta-actin were altered by some treatments. A higher molecular weight GAA protein corresponding to the 95 kD intermediate protein isoform was increased in the myogenic cells. Overall, there was a consistent increase in GAA protein expression after treatment with PMO 15.

We repeated PMO 13 and 15 treatments at least three times in all patient cell strains, except WM 8, independently seeded and transformed into myogenic cells, and consistently observed an increase in GAA activity (Fig. 3d). We did not perform biological replicates for WM 8 as this patient had an identical second mutation to WM 4, and the increase in GAA activity in a single experiment mirrored the activity observed in WM 4. In addition, we also performed myogenic conversion of both H 1 fibroblasts and nucleofected the cells with PMO 13, 15 and GTC. Nucleofection appeared to reduce GAA activity in the healthy control cells, but there was no further decrease in GAA activity after PMO 13 and 15 treatments.

## Discussion

There is a growing interest in the use of splice switching AOs as therapeutic agents, as new applications are identified and validated. The strategy outlined in this report will be relevant to the common c.-32-13T > G mutation, found in approximately two-thirds of adult and juvenile-onset Pompe patients. The infantile forms of GSD II arise from different mutations that result in minimal to undetectable levels of residual GAA activity (typically <1% of normal) and therefore would be unresponsive to the therapeutic strategy described here.

According to the Erasmus MC: Pompe disease *GAA* variant database (www.pompecenter.nl), over 500 causative GSD II mutations have been identified, and the phenotypes vary considerably. All nine patients’ cell strains used in this study carry the c.-32-13T > G mutation but differ in the nature of the mutation on the second allele, except WM 4 and 8. We showed that all patients have less than 40% of the GAA activity found in healthy control sample 2 that could be attributed to low levels of the full-length *GAA* transcript isoform retaining exon 2, despite the abnormal splicing induced by c.-32-13T > G mutation^32–37^.

We initially used patient-derived fibroblasts for the design and evaluation of AO sequences, as this cell type is frequently used for diagnosis^38^. However, it was necessary to evaluate the expression in myogenic cells as Pompe disease has a myogenic presentation, and also to ensure that any tissue-specific splicing does not influence PMO selection. Due to the variable nature of second mutations found in all patients^34–37,39,40^, any improvement after treatment with PMO 15 must be as a result of correct *GAA* transcript processing to include exon 2. Furthermore, the annealing site of PMO 15 is located within intron 1, and contains a strong transcriptional splicing repressor^10^. We also provide evidence for an increase in GAA protein expression and most importantly, an increase in GAA activity that must be regarded as a significant and appropriate readout for assessing treatment effect.

While we were performing this study, van der Wal *et al*.^25,26^ reported AO mediated splice correction of exon 2 through prevention of a pseudo-exon inclusion in the *GAA* transcript in two adult Pompe patients heterozygous for the c.-32-13T > G mutation. One patient possessed a second mutation with a frame-shifting deletion causing an early termination codon in exon 2, and the other patient had a missense mutation in exon 5. They undertook an initial AO screen using the *U7* snRNA expression system, while we used nucleofection to administer the PMOs. In another study, Pompe patient-derived myotubes were treated *in vitro* with a cocktail of three PMOs complementary to silencer motifs in exon 2 of the *GAA* transcript and showed evidence of reduced glycogen levels^27^.

We report unequivocal proof-of-concept for an AO mediated therapy applicable to a large subset of late-onset Pompe patients. Our study importantly provides evidence of a consistent increase of GAA activity in nine late-onset Pompe patient-derived cells carrying the c.-32-13T > G mutation. The responses were variable between the nine patients cell strains, attributed to various factors including cell quality, nature of the second mutation and perhaps more importantly, genetic differences in splicing machinery. Although some patients-derived cell strains responded to numerous AOs targeting different regions in our study, we were able to ascertain a single AO that could consistently increase GAA activity in all nine patients tested, regardless of the second mutation.

As with many other genetic therapies, one of the major concerns is uptake of the therapeutic compound into the target cell or tissue. Several options that may enhance AO uptake include different routes of administration, delivery regimens (dosage and frequency), AO backbone and chemistry, conjugation of the oligomer to cell-penetrating peptides^41–45^, aptamers^46^ and nanoparticles^47^. PMOs are known for their resistance to degradation in human serum, plasma and tissue extracts^48,49^. The main concern for the late-onset Pompe disease is proximal myopathy rather than cardiac^50^ and cognitive impairment^51^. The encouraging clinical outcomes reported on Eteplirsen/Exondys 51 and Golodirsen/Vyondys 53 for the treatment of Duchenne muscular dystrophy, supports further studies of this AO mediated therapy for late-onset Pompe disease.

In addition, a recent gene therapy study in non-human primates showed that delivering helper-dependent adenovirus expressing GAA to the liver produced sufficient secreted GAA for uptake by multiple muscles^52^. One advantage of using splice switching PMOs to address Pompe patients with c.-32-13T > G mutation is that when delivered through intravenous injections, these compounds accumulate in the liver and kidney^49^. Hence, the liver could produce and secrete GAA enzyme that may be taken up by muscle. Though it seems counterintuitive to address muscle disease by targeting the liver, a few studies have shown that liver-mediated GAA secretion is a possible therapy for Pompe disease^52–55^.

One question to be considered is whether PMO 15 should be evaluated in an animal model of the *GAA* c.-32-13T > G mutation before moving to clinical trials. Without doubt, the basic toxicology in two animal species should be undertaken, but to our knowledge, no available rodent model carries the human c.-32-13T > G mutation. Furthermore, we doubt whether a transgenic humanised rodent model would process the *GAA* transcript in the same manner as human cell, such that exon 2 was predominantly lost from the mature mRNA. The human and mouse genomic sequence homology in that region of the *GAA/ Gaa* is in the order of only 50%. Accordingly, we propose it may be possible to tread the path set for the first DMD exon skipping study reported by Kinali and colleagues^56^: an intramuscular injection into late-onset Pompe disease patients as a single-blind, placebo-controlled, dose-escalation, proof-of-concept study. Although our study was performed in MyoD induced primary fibroblast cells, uptake of PMO into muscle cells should not be a limitation since intramuscular injection of PMO in healthy mouse strains have shown efficient uptake by muscle cells^57^. From the many lessons learned, we hope that it does not take more than a decade to get a splice switching treatment for GSD II into the clinic.

## Methods

All cell culture reagents were purchased from Gibco, ThermoFisher Scientific, Scoresby, Australia and all chemicals from Sigma-Aldrich, Sydney, Australia unless otherwise stated. Sequences for all primers used in this study can be found in Table 3.

**Table 3:** Primers used in this study.

Name	Sequences (5′-3′)	Annealing Temp	Extension time	Cycle no.
**PCR (gDNA amplification)**				
intron 1F exon 2R	cagtctagacagcagggcaa agtaggatgtgccccaggag	55 °C	1 min	35
exon 16F exon 20R	caaggactctagcacctgg gaatctcccaagtcctgtga	60 °C	3 min	34
intron 14F exon 15R	catgctgggtggctgagaa ctcctcgtgtgtactacggc	60 °C	1 min	28
intron 5F intron 8R	cagagccctccaagtgaaga ttccaggaccaggtgacatc	60 °C	1 min	30
exon 10F intron 12R	actgccttccccgacttca ggagctttctgggatgag	60 °C	5 min	34
**RT-PCR (*GAA*** **transcript amplification)**				
exon 1F exon 5R	ggaaactgaggcacggagcg ggaccacatccatggcattgc	60 °C	2 min	34
exon 4F exon 10R	gtatatcacaggcctcgccg ctggtcatggaactcagcca	60 °C	2 min	34
exon 9F exon 17R	gggggttttcatcaccaacga ctgccaagggcctctactgg	60 °C	2 min	34
exon 16F exon 20R	caaggactctagcacctgg gaatctcccaagtcctgtga	60 °C	2 min	34
exon 4F exon 19R	gtatatcacaggcctcgccg gaagttggagacagggacacc	60 °C	3 min	35
**qPCR**				
*GAA* transcript (exon 1-2), amplification exon 1F(q) exon 1-2R(q)	tgggaaagctgaggttgtcg tcctacaggcccgctcc	Refer to Methods		
*TBP* transcript (exon 1-2), amplification exon 1-2F(q) exon 2R(q)	tctttgcagtgacccagcatcac cctagagcatctccagcacactct			

### Ethics approvals

The use of human cells was approved by Murdoch University Human Research Ethics Committee (approval 2013/156) and the Western Sydney Local Health District (WSLHD) Human Research Ethics Committee, Australia (approval HREC/17/WMEAD/358). Patient biopsies were collected after informed consent at the Westmead Hospital^58^. All samples were prepared and analysed in accordance with the protocols approved by the ethics committees of Murdoch University and WSLHD. All methods were performed in accordance with the relevant guidelines and regulations^58^.

### Cell culture

Pompe patients-derived fibroblasts GM00443 and GM11661, were purchased from Coriell Cell Repositories (Camden, New Jersey) and the remaining patients-derived fibroblasts were from Westmead Hospital, Sydney, Australia, obtained with informed consent (Approval HREC/17/WMEAD/358, SSA/17/WMEAD/392). Experiments were performed with Murdoch University Human Research Ethics Committee approval 2013/156. All cells were maintained in DMEM supplemented with L-Glutamine and 10% fetal bovine serum (FBS) (Scientifix, Cheltenham, Australia) at 37°C in a 5% CO_2_ atmosphere, except GM00443 which was maintained in 15% FBS, MEM. Normal human primary dermal fibroblasts were used as healthy controls.

### Nucleofection

All PMOs were synthesised by Sarepta Therapeutics (Cambridge, MA) with the exception of Gene Tools Control (GTC), which was purchased from Gene Tools, LLC (Philomath, OR). Nucleofection of fibroblasts was performed using the P3 Primary Cell 4D-nucleofector X kit S (32 RCT) (Lonza, Mt Waverley, Australia) and CA 137 program setting. Approximately 300,000 cells were nucleofected with various quantities of PMO (1, 0.5 and 0.05 nanomoles corresponding to 50, 25, 2.5 µM in the cuvettes) and incubated for 4 days before harvesting for RNA analysis and 6 days for protein analysis and GAA enzyme activity assessment. For the initial screening in fibroblasts, both transcript analysis and GAA activity were performed on day 4 samples.

### Adenoviral transduction and nucleofection

Patients-derived fibroblasts were nucleofected with PMO as described above and infected with MyoD expressing adenovirus, *Ad5.f50.AdApt.MyoD* (Native Antigen Company, Oxford, UK) at a multiplicity of infection of 200^31^, and plated at a density of 30,000 cells per 24 well pre-coated with Poly D-Lysine and Matrigel. The myogenic cells were allowed to differentiate in DMEM, low glucose, supplemented with 5% horse serum for 4 days before harvesting for RNA transcript studies or 6 days for protein analysis and GAA enzyme activity assessment.

### Genomic DNA extraction and PCR for sequencing

Genomic DNA was extracted using PureLink Genomic DNA mini kit (ThermoFisher Scientific) according to the manufacturer’s instructions. A total of 50 ng of DNA was amplified using *TaKaRa LA Taq* DNA polymerase with GC buffer II (Takara Bio USA, Inc., Clayton, Australia) using the primers listed in Table 3. The cycling conditions included 95°C for 5 min, 25–35 cycles of 95 °C 30 s, 55–60 °C 30 s and 72 °C 1–5 min. The cycling conditions are listed in Table 3. The PCR products were purified using Diffinity RapidTip and sequenced at the Australian Genome Research Facility (Perth, Australia) and compared to the reference *GAA* genomic sequence (Accession: NG_009822.1) using BLAST (https://blast.ncbi.nlm.nih.gov/Blast.cgi)^59^. All variants were submitted to ClinVar (SUB6437945; SUB6703839).

### RT-PCR and qPCR

Total RNA was extracted using MagMax nucleic acid isolation kits (ThermoFisher Scientific) according to the manufacturer’s instructions with a DNAse step included in the kit. Approximately 125 ng of total RNA, assessed using the Nanodrop (ND-1000, ThermoFisher Scientific) for quality and quantity, was used to synthesise cDNA using SuperScript IV reverse transcriptase (ThermoFisher Scientific) with 200 ng random hexamers (ThermoFisher Scientific) in a 20 µl reaction. Transcripts were amplified using 0.5 µl of cDNA as the template and *TaKaRa LA Taq* DNA polymerase with GC buffer II (Takara Bio USA). *GAA* transcript was amplified using the primers and cycling conditions listed in Table 3. General cycling reactions included 95 °C for 5 min, 34–35 cycles of 95 °C 30 s, 60 °C 30 s and 72 °C 2–3 min. The PCR products were fractionated on 2% agarose gels in Tris-Acetate-EDTA buffer. The qPCR reactions were performed using fast SYBR Green (ThermoFisher Scientific) and 100 nM (96.5% primer efficiency) and 500 nM (104.9% primer efficiency) primers for *GAA* and *TBP* transcript, respectively^58^. Reactions were performed in a CFX384 Touch Real-Time PCR detection system (Bio-Rad Laboratories Pty., Ltd., Gladesville, Australia) and *GAA* (Accession: NM_000152.4) transcript expression relative to the reference transcript *TBP* (Accession: NM_003194.4) was calculated. The cycling reactions included 95 °C for 1 min, 39 cycles of 95 °C 3 s, 60 °C 15 s and 72 °C 30 s. The results were analysed on the Bio-Rad CFX Manager Software Version 3.1 (Bio-Rad Laboratories Pty., Ltd., Gladesville, Australia; https://www.bio-rad.com/en-au/product/previous-qpcr-software-releases?ID=OO2BB34VY). The relative expression of *GAA* to *TBP* mRNA was determined using the 2^−ΔΔCT^ method^60^ and presented as a fold change compared to the healthy or Gene Tools Control treated patients-derived fibroblasts.

### GAA enzyme activity assay

Cells were subjected to three freeze-thaw cycles before resuspension in 50 µl of lysis buffer^61^ (10 mM HEPES, 70 mM sucrose, 220 mM mannitol supplemented with 1 × protease inhibitors) and sonicated 6 times for 1 s. The cell lysate was centrifuged at 11,000 × g for 10 min at 4°C, the supernatant was collected and total protein concentration was measured using Pierce BCA protein assay kit (ThermoFisher Scientific). Approximately 3–5 µg of the total protein lysate in 10 µl volume was used for enzymatic reactions under two pH conditions, 3.9 (for acid-α-glucosidase activity) and 6.5 (for neutral α-glucosidase activity). GAA enzyme activity was initiated by adding 20 µl of 1.4 mM artificial substrate 4-Methylumbelliferyl-β-D-glucopyranoside (4-MUG) prepared in two 0.2 M acetate buffers, pH 3.9 and pH 6.5. The reaction was incubated at 37 °C for 1 h before adding 200 µl of stop buffer (0.5 M sodium carbonate, pH 10.7)^58^. The fluorescent signals were measured using FLUOstar Omega (BMG LABTECH, Mornington, Australia) with 355 nm excitation and 460 nm emission filters. The ratio of signals generated at pH 3.9 to those at pH 6.5 was calculated, and the results were expressed as a fold change, compared to the untreated or Gene Tools Control treated patients-derived fibroblasts. All assays were performed in triplicate.

### Western blotting

Approximately 4 µg of protein, as determined by a BCA assay, was loaded onto NuPAGE Novex 4–12% BIS/Tris gels (ThermoFisher Scientific) and fractionated at 200 volts until the bromophenol blue marker was at the bottom of the gel. Gel contents were then transferred onto Pall FluoroTrans membranes (Fisher Biotec Wembley, Australia) and probed with rabbit anti-GAA antibody (Abcam, cat. no. 137068, Melbourne, Australia)^26^ at 1:1,000 dilution, anti-desmin antibody (ThermoFisher Scientific, cat. no. PA5-16705)^62^ at 1:5,000 dilution, mouse monoclonal anti-β-tubulin antibody (DSHB, cat. no. E7, Iowa City, Iowa)^63^ at 1:2,000–6,000 dilution and mouse monoclonal anti-β-actin antibody (Sigma-Aldrich, cat. no. A5316)^64^ at 1:100,000 dilution overnight at 4 °C. Polyclonal goat anti-rabbit or anti-mouse immunoglobulins/HRP (Dako, cat. no P0448 and D0447 respectively, North Sydney, Australia) at a dilution of 1:10,000 and Luminata Crescendo Western HRP substrate (Merk Millipore, Bayswater, Australia) were used for immunodetection and a serial scan of 30 s was performed using Fusion FX system (Vilber Lourmat, Marne-la-Vallée, France). The entire image was processed and densitometric analysis was performed using ImageJ (NIH; https://imagej.nih.gov/ij/download.html)^65^.

### Statistical analysis

One-way ANOVA with Bonferroni correction was performed to determine significance (p < 0.05).

## Supplementary information

Supplementary material.

## Data Availability

All data generated or analysed during this study are included in this published article (and its Supplementary Information file).

## References

[1] Chillon M (1995). Mutations in the cystic fibrosis gene in patients with congenital absence of the vas deferens. N. Engl. J. Med..

[2] Spritz RA (1981). Base substitution in an intervening sequence of a beta+-thalassemic hu man globin gene. Pro.c Natl. Acad. Sci. USA.

[3] Kim J (2019). Patient-Customized Oligonucleotide Therapy for a Rare Genetic Disease. N Engl J Med.

[4] FDA grants accelerated approval to first targeted treatment for rare Duchenne muscular dystrophy mutation, Available online, https://www.fda.gov/news-events/press-announcements/fda-grants-accelerated-approval-first-targeted-treatment-rare-duchenne-muscular-dystrophy-mutation (2019).

[5] FDA grants accelerated approval to first drug for Duchenne muscular dystrophy [(accessed on 19 September 2016], Available online, http://www.fda.gov/NewsEvents/Newsroom/PressAnnouncements/ucm521263.htm (2016).

[6] Ottesen EW (2017). ISS-N1 makes the First FDA-approved Drug for Spinal Muscular Atrophy. Transl. Neurosci..

[7] Huie ML (1994). Aberrant splicing in adult onset glycogen storage disease type II (GSDII): molecular identification of an IVS1 (-13T- > G) mutation in a majority of patients and a novel IVS10 (+1GT->CT) mutation. Hum. Mol. Genet..

[8] Bergsma AJ (2019). A genetic modifier of symptom onset in Pompe disease. EBioMedicine.

[9] Boerkoel CF (1995). Leaky splicing mutation in the acid maltase gene is associated with delayed onset of glycogenosis type II. Am. J. Hum. Genet..

[10] Raben N, Nichols RC, Martiniuk F, Plotz PH (1996). A model of mRNA splicing in adult lysosomal storage disease (glycogenosis type II). Hum. Mol. Genet..

[11] Mehler M, DiMauro S (1977). Residual acid maltase activity in late-onset acid maltase deficiency. Neurology.

[12] Umapathysivam K, Hopwood JJ, Meikle PJ (2005). Correlation of acid alpha-glucosidase and glycogen content in skin fibroblasts with age of onset in Pompe disease. Clin. Chim. Acta..

[13] van der Ploeg AT, Reuser AJ (2008). Pompe’s disease. Lancet.

[14] Cupler EJ (2012). Consensus treatment recommendations for late-onset Pompe disease. Muscle Nerve.

[15] Stepien KM, Hendriksz CJ, Roberts M, Sharma R (2016). Observational clinical study of 22 adult-onset Pompe disease patients undergoing enzyme replacement therapy over 5years. Mol. Genet. Metab..

[16] Schoser B (2017). Survival and long-term outcomes in late-onset Pompe disease following alglucosidase alfa treatment: a systematic review and meta-analysis. J. Neurol..

[17] Lachmann, R. H. Treating Lysosomal Storage Disorders: What Have We Learnt? *J Inherit Metab Dis*, 10.1002/jimd.12131 (2019). 10.1002/jimd.12131 31140601

[18] Zhou Q (2011). Strategies for Neoglycan conjugation to human acid alpha-glucosidase. Bioconjug Chem.

[19] Pena LDM (2019). Safety, tolerability, pharmacokinetics, pharmacodynamics, and exploratory efficacy of the novel enzyme replacement therapy avalglucosidase alfa (neoGAA) in treatment-naive and alglucosidase alfa-treated patients with late-onset Pompe disease: A phase 1, open-label, multicenter, multinational, ascending dose study. Neuromuscul Disord.

[20] Smith BK (2013). Phase I/II trial of adeno-associated virus-mediated alpha-glucosidase gene therapy to the diaphragm for chronic respiratory failure in Pompe disease: initial safety and ventilatory outcomes. Hum. Gene Ther..

[21] Ronzitti G, Collaud F, Laforet P, Mingozzi F (2019). Progress and challenges of gene therapy for Pompe disease. Ann Transl Med.

[22] Arechavala-Gomeza V (2007). Comparative analysis of antisense oligonucleotide sequences for targeted skipping of exon 51 during dystrophin pre-mRNA splicing in human muscle. Hum Gene Ther.

[23] Zhou H (2013). A novel morpholino oligomer targeting ISS-N1 improves rescue of severe spinal muscular atrophy transgenic mice. Hum Gene Ther.

[24] Mitrpant C (2013). Improved antisense oligonucleotide design to suppress aberrant SMN2 gene transcript processing: towards a treatment for spinal muscular atrophy. PLoS One.

[25] van der Wal E, Bergsma AJ, Pijnenburg JM, van der Ploeg AT, Pijnappel WP (2017). Antisense Oligonucleotides Promote Exon Inclusion and Correct the Common c.-32-13T>G GAA Splicing Variant in Pompe Disease. Mol. Ther. Nucleic Acids.

[26] van der Wal E (2017). GAA Deficiency in Pompe Disease Is Alleviated by Exon Inclusion in iPSC-Derived Skeletal Muscle Cells. Mol. Ther. Nucleic Acids.

[27] Goina E, Peruzzo P, Bembi B, Dardis A, Buratti E (2017). Glycogen Reduction in Myotubes of Late-Onset Pompe Disease Patients Using Antisense Technology. Mol. Ther..

[28] Mendell JR (2013). Eteplirsen for the Treatment of Duchenne Muscular Dystrophy. Annals. of Neurology.

[29] Mendell JR (2013). Results at 2 years of a phase IIb extension study of the exon-skipping drug eteplirsen in patients with DMD. Neuromuscular Disord..

[30] Piva F, Giulietti M, Nocchi L, Principato G (2009). SpliceAid: a database of experimental RNA target motifs bound by splicing proteins in humans. Bioinformatics.

[31] Lattanzi L (1998). High efficiency myogenic conversion of human fibroblasts by adenoviral vector-mediated MyoD gene transfer. An alternative strategy for *ex vivo* gene therapy of primary myopathies. J. Clin. Invest..

[32] McCready ME (2007). Development of a clinical assay for detection of GAA mutations and characterization of the GAA mutation spectrum in a Canadian cohort of individuals with glycogen storage disease, type II. Mol Genet Metab.

[33] Kroos M (2008). Update of the Pompe disease mutation database with 107 sequence variants and a format for severity rating. Hum Mutat.

[34] Joshi PR (2008). Molecular diagnosis of German patients with late-onset glycogen storage disease type II. J Inherit Metab Dis.

[35] Hermans MM (2004). Twenty-two novel mutations in the lysosomal alpha-glucosidase gene (GAA) underscore the genotype-phenotype correlation in glycogen storage disease type II. Hum Mutat.

[36] Ausems MG (1996). Homozygous deletion of exon 18 leads to degradation of the lysosomal alpha-glucosidase precursor and to the infantile form of glycogen storage disease type II. Clin. Genet..

[37] Zhong N, Martiniuk F, Tzall S, Hirschhorn R (1991). Identification of a missense mutation in one allele of a patient with Pompe disease, and use of endonuclease digestion of PCR-amplified RNA to demonstrate lack of mRNA expression from the second allele. Am J Hum Genet.

[38] Disease AWGoMoP (2006). Pompe disease diagnosis and management guideline. Genet Med.

[39] Palmer RE (2007). Pompe disease (glycogen storage disease type II) in Argentineans: clinical manifestations and identification of 9 novel mutations. Neuromuscul Disord.

[40] Wang QL (2016). Phenotypes and Genotypes in Five Children with Congenital Insensitivity to Pain with Anhidrosis. Pediatr Neurol.

[41] Betts C (2012). Pip6-PMO, A New Generation of Peptide-oligonucleotide Conjugates With Improved Cardiac Exon Skipping Activity for DMD Treatment. Mol. Ther. Nucleic Acids.

[42] Hammond SM (2016). Systemic peptide-mediated oligonucleotide therapy improves long-term survival in spinal muscular atrophy. Proc. Natl. Acad. Sci. USA.

[43] McClorey G, Moulton HM, Iversen PL, Fletcher S, Wilton SD (2006). Antisense oligonucleotide-induced exon skipping restores dystrophin expression *in vitro* in a canine model of DMD. Gene Ther..

[44] Moulton HM, Nelson MH, Hatlevig SA, Reddy MT, Iversen PL (2004). Cellular uptake of antisense morpholino oligomers conjugated to arginine-rich peptides. Bioconjug Chem.

[45] McClorey G (2006). Induced dystrophin exon skipping in human muscle explants. Neuromuscul Disord.

[46] Subramanian N, Kanwar JR, Kanwar RK, Krishnakumar S (2015). Targeting Cancer Cells Using LNA-Modified Aptamer-siRNA Chimeras. Nucleic Acid Ther..

[47] Falzarano MS, Passarelli C, Ferlini A (2014). Nanoparticle delivery of antisense oligonucleotides and their application in the exon skipping strategy for Duchenne muscular dystrophy. Nucleic Acid Ther..

[48] Hudziak RM (1996). Resistance of morpholino phosphorodiamidate oligomers to enzymatic degradation. Antisense Nucleic Acid Drug Dev.

[49] Amantana A (2007). Pharmacokinetics, biodistribution, stability and toxicity of a cell-penetrating peptide-morpholino oligomer conjugate. Bioconjug. Chem..

[50] Limongelli G, Fratta F (2011). S1.4 Cardiovascular involvement in Pompe disease. Acta Myol.

[51] Bekircan-Kurt CE (2017). New mutations and genotype-phenotype correlation in late-onset Pompe patients. Acta Neurol Belg.

[52] Rastall DP (2016). Long-term, high-level hepatic secretion of acid alpha-glucosidase for Pompe disease achieved in non-human primates using helper-dependent adenovirus. Gene. Ther..

[53] Han SO (2017). Low-Dose Liver-Targeted Gene Therapy for Pompe Disease Enhances Therapeutic Efficacy of ERT via Immune Tolerance Induction. Mol. Ther. Methods Clin. Dev..

[54] Raben N (2001). Conditional tissue-specific expression of the acid alpha-glucosidase (GAA) gene in the GAA knockout mice: implications for therapy. Hum. Mol. Genet..

[55] Rastall DP (2016). Long-term, high-level hepatic secretion of acid alpha-glucosidase for Pompe disease achieved in non-human primates using helper-dependent adenovirus. Gene Ther..

[56] Kinali M (2009). Local restoration of dystrophin expression with the morpholino oligomer AVI-4658 in Duchenne muscular dystrophy: a single-blind, placebo-controlled, dose-escalation, proof-of-concept study. Lancet Neurol..

[57] Cao L, Han G, Gu B, Yin H (2014). Wild-type mouse models to screen antisense oligonucleotides for exon-skipping efficacy in Duchenne muscular dystrophy. PLoS One.

[58] Aung-Htut, M. T., Ham, K. A., Tchan, M. C., Fletcher, S. & Wilton, S. D. Novel Mutations Found in Individuals with Adult-Onset Pompe Disease. *Genes**(**Basel**)* **11**, 10.3390/genes11020135 (2020). 10.3390/genes11020135 PMC7073677 32012848

[59] Altschul SF, Gish W, Miller W, Myers EW, Lipman DJ (1990). Basic local alignment search tool. J Mol Biol.

[60] Ganger MT, Dietz GD, Ewing SJ (2017). A common base method for analysis of qPCR data and the application of simple blocking in qPCR experiments. BMC Bioinformatics.

[61] Nilsson MI (2012). Aerobic training as an adjunctive therapy to enzyme replacement in Pompe disease. Mol. Genet. Metab..

[62] Chen L (2012). HSCs play a distinct role in different phases of oval cell-mediated liver regeneration. Cell Biochem Funct.

[63] Hsu J (2019). E2F4 regulates transcriptional activation in mouse embryonic stem cells independently of the RB family. Nat Commun.

[64] Bunnell TM, Burbach BJ, Shimizu Y, Ervasti JM (2011). Beta-Actin specifically controls cell growth, migration, and the G-actin pool. Mol Biol Cell.

[65] Schneider CA, Rasband WS, Eliceiri KW (2012). NIH Image to ImageJ: 25 years of image analysis. Nat Methods.

