# Supplementary material for: Splice modulating antisense oligonucleotides restore some acid-alpha-glucosidase activity in cells derived from patients with late-onset Pompe disease
Source: Sci Rep. 2020 Apr 21;10:6702. doi: 10.1038/s41598-020-63461-2 (PMC7174337; doi:10.1038/s41598-020-63461-2)
Supplement: Supplementary file 1 — Supplementary material. [file 41598_2020_63461_MOESM1_ESM.pdf]

Splice modulating antisense oligonucleotides restore some acid-alpha-glucosidase activity in cells derived from patients with late-onset Pompe disease

May Thandar Aung-Htut,[1,5,+] Kristin A. Ham,[1,5,+] Michel Tchan,[2,3] Russell Johnsen,[1] Frederick J. Schnell,[4] Sue Fletcher,[1,5,*] Steve D Wilton[1,5,*]

[1]Centre for Molecular Medicine and Innovative Therapeutics, Murdoch University, Murdoch, 6150, Australia.

[2]Genetic Medicine, Westmead Hospital, Sydney, 2145, Australia

[3]Sydney Medical School, The University of Sydney, Sydney, 2006, Australia.

[4]Sarepta Therapeutics, Cambridge, 02142, USA.

[5]Perron Institute for Neurological and Translational Science and Centre for Neuromuscular and Neurological Disorders, The University of Western Australia, Perth, 6009, Australia.

[*]corresponding senior authors S.F. and S.D.W.: s.wilton@murdoch.edu.au

[+]these authors contributed equally

Short title: Splice switching therapy for Pompe disease

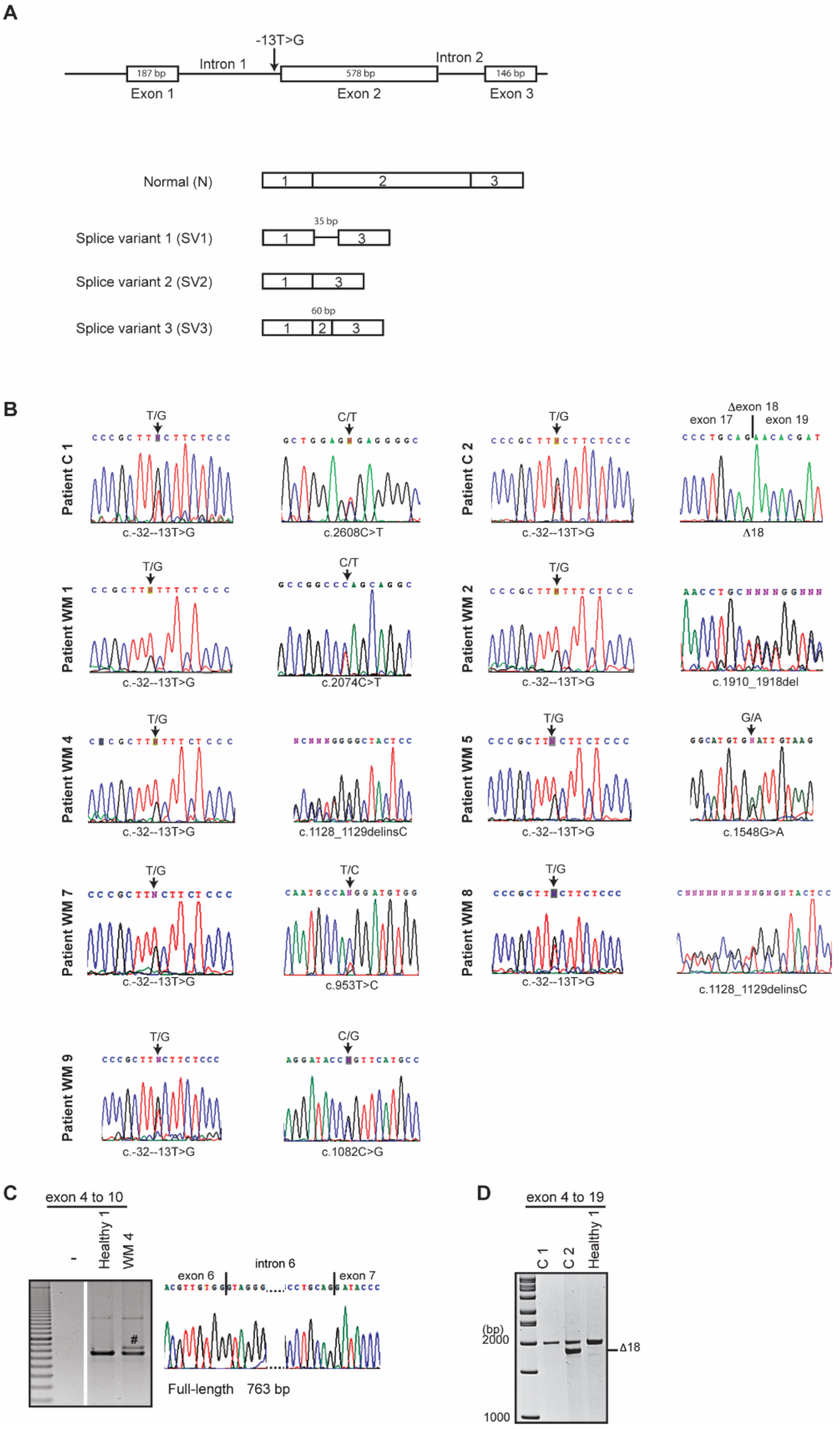

Figure S1. (A) *GAA* transcript variant isoforms generated by the c.-32-13T>G mutation. (B) Chromatograms of Sanger sequencing to identify mutations for each patient. Patient ID are shown on the left. (C) RT-PCR products of *GAA* transcripts amplified from exon 4 to 10 using RNA isolated from patient WM 4 and healthy 1 fibroblasts. Chromatogram of Sanger sequencing showing intron 6 inclusion in the transcript variant (#) was shown on the right. (D) RT-PCR products of *GAA* transcripts (exon 4 to 19) amplified from RNA isolated from patient C 1, C 2 and healthy fibroblasts confirmed deletion of exon 18 in patient C 2 *GAA* transcript. Chromatogram for Sanger sequencing is shown in (B).

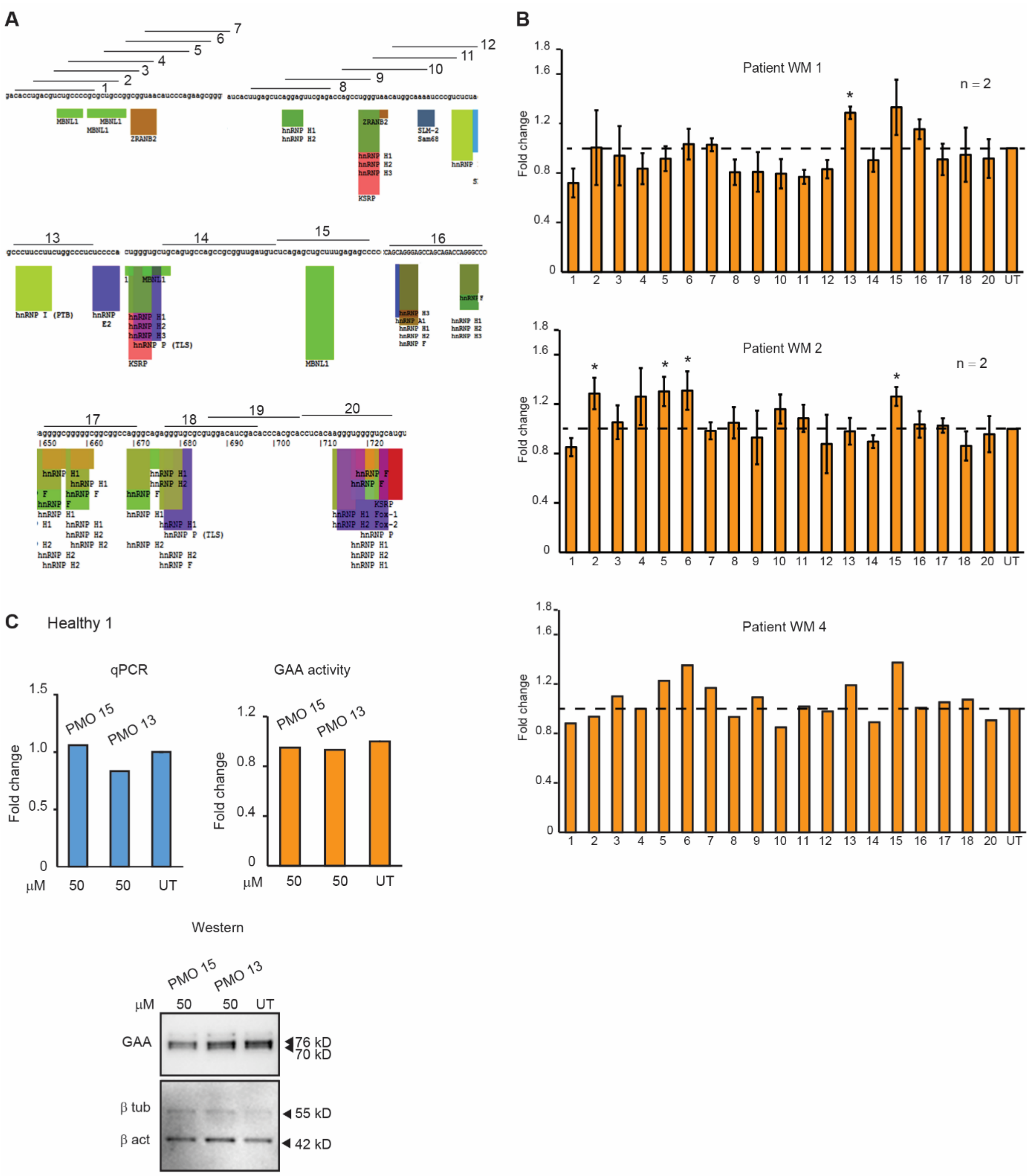

Figure S2. (A) Potential splicing silencer regions identified by Splice Aid 1.0 and the splice modulating PMOs designed to enhance exon 2 inclusion in the *GAA* transcript. The PMO numbers are shown. (B) The improvement in GAA activity in treated compared to untreated (UT) patient WM 1, 2 and 4 derived fibroblasts, presented as a fold change. n=2 (biological replicates except WM 4). Error bars; SD.* $p<0.05$ (ANOVA with Bonferroni correction, compared to UT). (C) qPCR, GAA activity and western analysis of healthy 1 fibroblasts treated with PMO 15 and 13. UT; untreated. The gels were cropped for presentation, and full-length gels are presented in Supplementary Fig. S5.

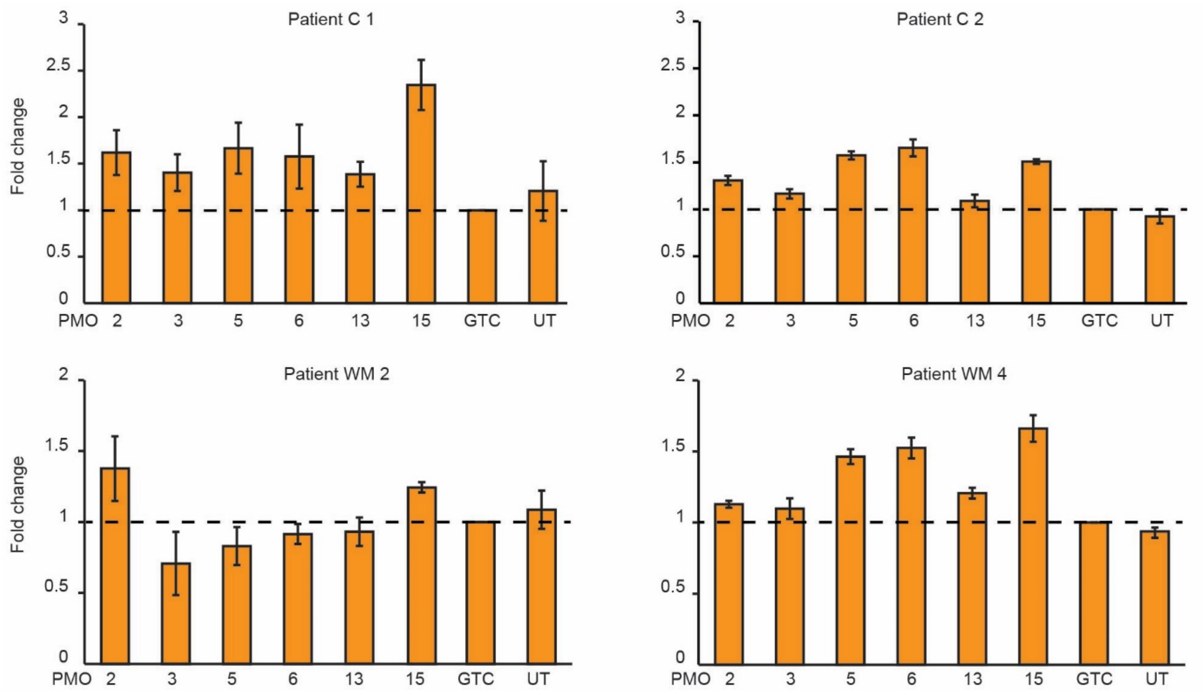

Figure S3. GAA activity of patient C 1, 2, WM 2 and 4 forced myogenic cells after treatment with various PMOs, GTC (Gene Tools control) and untreated (UT). Data represent the mean ±SD of triplicates.

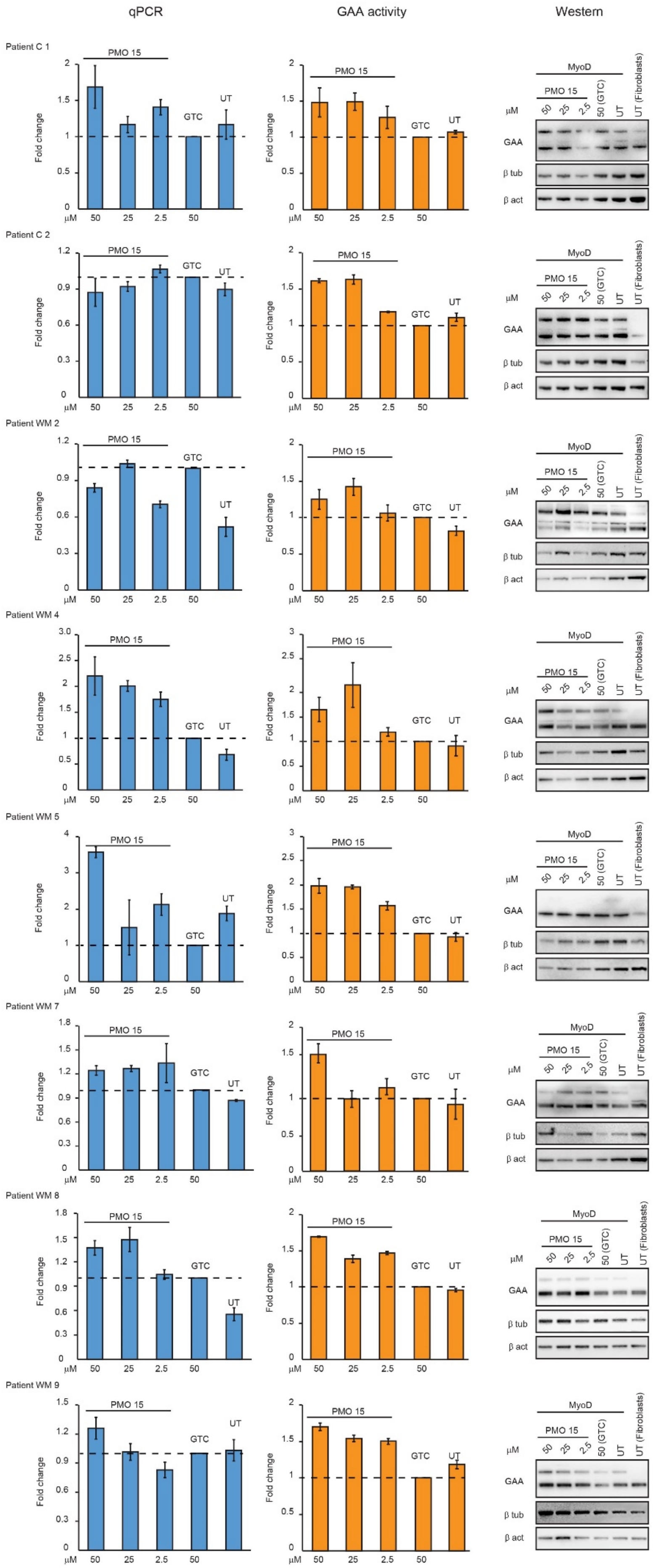

qPCR
GAA activity
Western
Patient C 1
Patient C 2
Patient WM 2
Patient WM 4
Patient WM 5
Patient WM 7
Patient WM 8
Patient WM 9
PMO 15
GTC
UT
Fold change
μM
50
25
2.5
MyoD
50 (GTC)
UT (Fibroblasts)
GAA
β tub
β act

Figure S4. qPCR analysis of *GAA* transcript with exon 2, GAA activity and protein in patient C 1, C 2, WM 2, WM 4, WM 5, WM 7, WM 8 and 9 forced myogenic cells after treatment with various dosages of PMO 15, GTC (Gene Tools control) and untreated (UT). Data represent the mean ±SEMs of triplicates. H 1; healthy 1, H 2; healthy 2, β tub; beta-tubulin, β act; beta-actin. The gels were cropped for presentation, and full-length gels are presented in Supplementary Fig. S5.

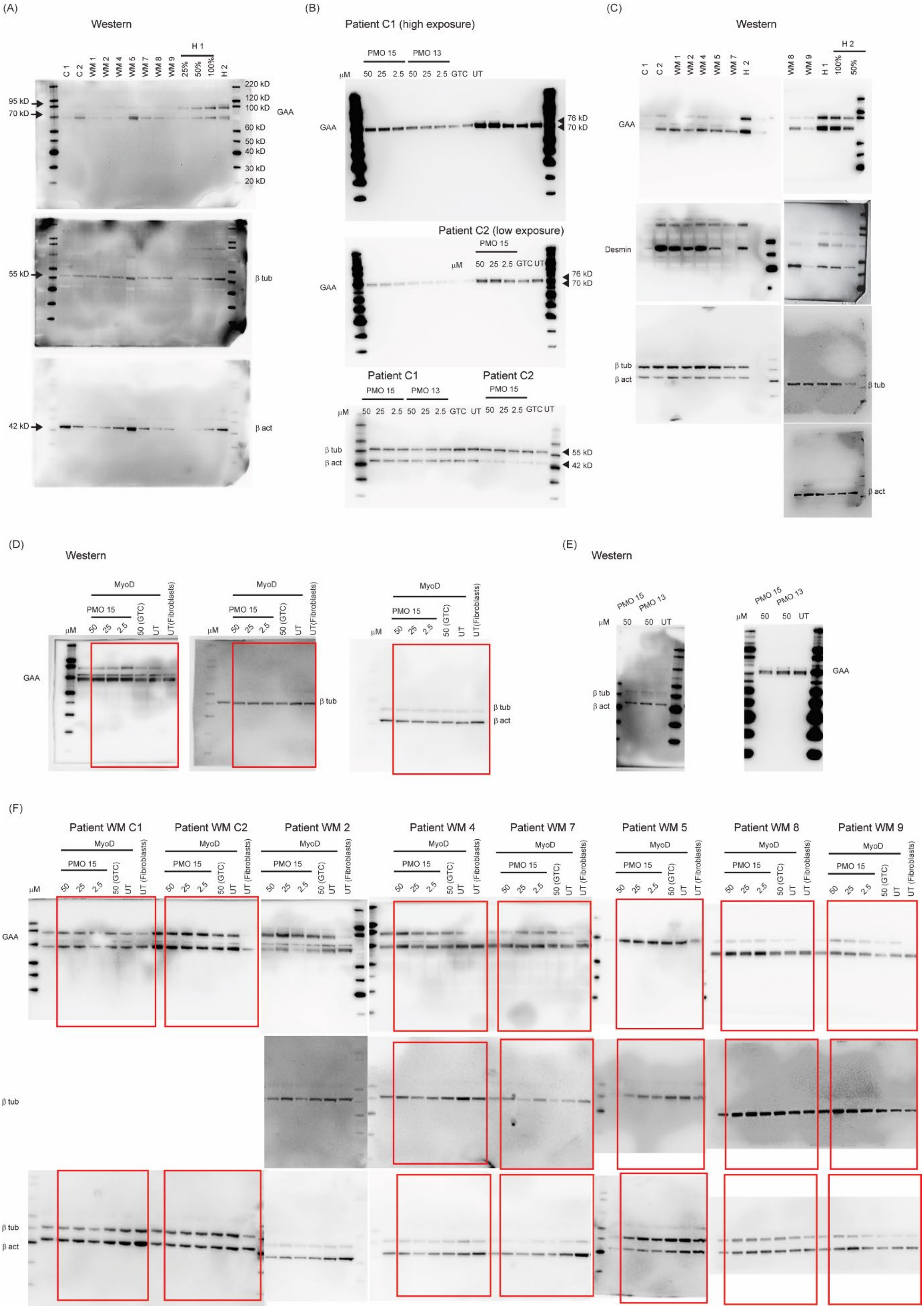
(A)
Western
H 1
C 1
C 2
WM 1
WM 2
WM 4
WM 5
WM 7
WM 8
WM 9
25%
50%
100%
H 2
220 kD
120 kD
100 kD
60 kD
50 kD
40 kD
30 kD
20 kD
95 kD
70 kD
GAA
55 kD
β tub
42 kD
β act
(B)
Patient C1 (high exposure)
PMO 15
PMO 13
μM
50 25 2.5 50 25 2.5 GTC UT
GAA
76 kD
70 kD
Patient C2 (low exposure)
PMO 15
μM
50 25 2.5 GTC UT
GAA
76 kD
70 kD
Patient C1
Patient C2
PMO 15
PMO 13
PMO 15
μM
50 25 2.5 50 25 2.5 GTC UT 50 25 2.5 GTC UT
β tub
β act
55 kD
42 kD
(C)
Western
C 1
C 2
WM 1
WM 2
WM 4
WM 5
WM 7
H 2
WM 8
WM 9
H 1
H 2
100%
50%
GAA
Desmin
β tub
β act
β tub
β act
(D)
Western
MyoD
PMO 15
μM
50 25 2.5 50 (GTC) UT UT(Fibroblasts)
GAA
β tub
β tub
β act
(E)
Western
PMO 15
PMO 13
μM
50 50 UT
β tub
β act
GAA
(F)
Patient WM C1
Patient WM C2
Patient WM 2
Patient WM 4
Patient WM 7
Patient WM 5
Patient WM 8
Patient WM 9
MyoD
PMO 15
μM
50 25 2.5 50 (GTC) UT UT (Fibroblasts)
GAA
β tub
β tub
β act

Figure S5. Full blots for (A) Figure 1C, (B) Figure 2C, (C) Figure 3A and (D) Figure 3 C (E) Figure S2 and (F) Figure S4.
